# Variability in Pediatric Infectious Disease Consultants' Recommendations for Management of Community-Acquired Pneumonia

**DOI:** 10.1371/journal.pone.0020325

**Published:** 2011-05-31

**Authors:** Adam L. Hersh, Daniel J. Shapiro, Jason G. Newland, Philip M. Polgreen, Susan E. Beekmann, Samir S. Shah

**Affiliations:** 1 Pediatric Infectious Diseases, University of Utah, Salt Lake City, Utah, United States of America; 2 Institute for Health Policy Studies, University of California San Francisco, San Francisco, California, United States of America; 3 Children's Mercy Hospital and Clinics, Kansas City, Missouri, United States of America; 4 University of Iowa Carver College of Medicine, Iowa City, Iowa, United States of America; 5 Departments of Pediatrics and Biostatistics and Epidemiology, and the Center for Clinical Epidemiology and Biostatistics, University of Pennsylvania School of Medicine, Philadelphia, Pennsylvania, United States of America; 6 Division of Infectious Diseases, Center for Pediatric Clinical Effectiveness, The Children's Hospital of Philadelphia, Philadelphia, Pennsylvania, United States of America; Los Angeles Biomedical Research Institute, United States of America

## Abstract

**Background:**

Community-acquired pneumonia (CAP) is a common childhood infection. CAP complications, such as parapneumonic empyema (PPE), are increasing and are frequently caused by antibiotic-resistant organisms. No clinical guidelines currently exist for management of pediatric CAP and no published data exist about variations in antibiotic prescribing patterns. Our objectives were to describe variation in CAP clinical management for hospitalized children by pediatric infectious disease consultants and to examine associations between recommended antibiotic regimens and local antibiotic resistance levels.

**Methods:**

We surveyed pediatric members of the Emerging Infections Network, which consists of 259 pediatric infectious disease physicians. Participants responded regarding their recommended empiric antibiotic regimens for hospitalized children with CAP with and without PPE and their recommendations for duration of therapy. Participants also provided information about the prevalence of penicillin non-susceptible *S. pneumoniae* and methicillin-resistant *S. aureus* (MRSA) in their community.

**Results:**

We received 148 responses (57%). For uncomplicated CAP, respondents were divided between recommending beta-lactams alone (55%) versus beta-lactams in combination with another class (40%). For PPE, most recommended a combination of a beta-lactam plus an anti-MRSA agent, however, they were divided between clindamycin (44%) and vancomycin (57%). The relationship between reported antibiotic resistance and empiric regimen was mixed. We found no relationship between aminopenicillin use and prevalence of penicillin non-suscepetible *S. pneumonia*e or clindamycin use and clindamycin resistance, however, respondents were more likely to recommend an anti-MRSA agent when MRSA prevalence increased.

**Conclusions:**

Substantial variability exists in recommendations for CAP management. Development of clinical guidelines via antimicrobial stewardship programs and dissemination of data about local antibiotic resistance patterns represent opportunities to improve care.

## Introduction

Community acquired pneumonia (CAP) is a common serious infection in childhood, accounting for over 150,000 hospitalizations each year in the US [1]. Despite its importance, no national clinical guidelines currently exist for the management of pediatric CAP. Recent evidence indicates that overall antibiotic utilization for hospitalized children varies widely across hospitals [2], although the extent to which this variation exists for individual conditions, such as CAP, remains unknown.

CAP-associated complications, such parapneumonic empyema (PPE), have increased in recent years [1], [3], [4]. Antibiotic-resistant organisms, especially *Staphylococcus aureus* and *Streptococcus pneumoniae* are important causes of CAP and PPE. Resistance patterns for these organisms vary widely throughout the United States [5], [6], [7], [8]. The extent to which antibiotic recommendations for pneumonia reflect local resistance patterns for these organisms is unknown. For instance, it is unknown whether clindamycin and vancomycin, which have activity against methicillin-resistant *S. aureus* (MRSA), are recommended more frequently for PPE in communities where MRSA prevalence is higher. Delays in matching the antibiotic spectrum to the causative bacteria is clinically relevant for CAP [9], [10].

Infectious disease consultants are frequently involved in the care of children with CAP and local guideline development for the management of CAP. Additionally, interventions such as antimicrobial stewardship programs (ASPs, hospital-based interventions where antimicrobial prescribing is monitored and physicians receive feedback), and unit- or disease-specific antibiograms provide assistance to physicians in selecting the optimal empiric antibiotic and are typically led by infectious disease consultants [11], [12], [13], [14]. The objectives of this study were to describe variation in the clinical management by pediatric infectious diseases consultants for hospitalized children with CAP and to examine the associations between infectious disease consultants' recommended antibiotic regimens, reported antibiotic resistance levels in their communities, and the presence of ASPs.

## Materials and Methods

### Ethics Statement

This study was determined to be exempt from review by the University of Iowa Institutional Review Board and is not considered to be human subjects research.

### Study design

We conducted a survey of pediatric infectious diseases consultants during Fall 2009 regarding their management recommendations for pediatric CAP and the reported prevalence of antibiotic resistance among *S. pneumoniae* and *S. aureus* in their community. All survey questions related to hospitalized children ages 1–18 years, with no known underlying medical conditions predisposing them to severe or recurrent pneumonia. Respondents completed an electronic or paper data entry form. Non-respondents received up to 2 follow-up queries.

### Data Source

The data source was the pediatric members of the Emerging Infections Network which consists of 259 pediatric infectious diseases physicians throughout North America. Membership is drawn from the Pediatric Infectious Diseases Society and the Infectious Diseases Society of America and includes physicians from 44 states and 3 Canadian Provinces. EIN members represent over 50% of the children's hospitals in the United States [15].

### Survey Questions

The EIN maintains demographic data on individual members including years of practice, geographic region and practice setting. For this survey we ascertained supplementary information including the type of hospital with which the respondent is primarily affiliated (freestanding children's hospital, children's hospital within a hospital and general hospital with pediatric beds) and whether or not the hospital has housestaff, whether the hospital has an antimicrobial stewardship program, and whether there is a clinical guideline or pathway for CAP.

To address the first objective, survey respondents were asked to select the specific antibiotic agent or combination of agents that they recommended for children hospitalized for CAP, distinguishing between uncomplicated and PPE cases. We defined uncomplicated CAP by the presence of a focal consolidation and PPE by the presence of a focal consolidation plus empyema. For each case, respondents selected among one or more of the following agents: ampicillin, ampicillin/sulbactam, 2^nd^/3^rd^ generation cephalosporins, azithromycin, vancomycin, clindamycin and linezolid. For subsequent analyses, we categorized ampicillin, ampicillin/sulbactam and cephalosporins as beta-lactam antibiotics and vancomycin, clindamycin and linezolid as anti-MRSA antibiotics. Respondents were also asked to select the duration of antibiotic therapy they recommend for uncomplicated and PPE cases. Respondents selected among the following categories: 3–5 days; 6–7 days; 8–10 days; 11–14 days; 15–21 days; >21 days.

To collect data for the second objective, respondents were asked to estimate the resistance levels among isolates in their hospital for *S. pneumoniae* and *S. aureus*. To characterize *S. pneumoniae*, they indicated the percentage of isolates with intermediate susceptibility to penicillin (defined as mean inhibitory concentration (MIC) of 4 µg/mL) and penicillin resistance (defined as an MIC of ≥8 µg/mL). To characterize *S. aureus*, they indicated the percentage of all *S. aureus* isolates that are MRSA and the percentage of MRSA isolates that are resistant to clindamycin (including by D-test). For all three estimates, they selected among the following categories: <10%; 10–25%; 26–50%; and >50%.

### Analysis

We used descriptive statistics to describe variations in the recommended empiric antibiotic regimens, andrecommended treatment durations. We performed a chi-square test for linear trend to determine whether there was an association between respondents' recommended antibiotic regimens and their estimates for resistance levels. Specifically, we hypothesized that respondents that reported a lower percentage of penicillin non-susceptible isolates (intermediate plus resistant) would be more likely to recommend an aminopenicillin-containing antibiotic (e.g. ampicillin or ampicillin/sulbactam) in their empiric regimen for uncomplicated CAP. We also hypothesized that respondents reporting a higher reported percentage of MRSA among *S. aureus* isolates would be more likely to include an anti-MRSA agent in their empiric regimen for PPE. Additionally, we hypothesized that among those who included an anti-MRSA agent for PPE, those with higher reported clindamycin resistance would be less likely to select clindamycin (as opposed to vancomycin or linezolid).

Because any observed relationships between antibiotic regimens and reported resistance levels could be subject to confounding, we conducted multivariable analysis using logistic regression to examine the association of other factors, besides resistance, with antibiotic recommendations using data from the survey. Specifically, we developed a model for the relationship between reported resistance for *S. pneumoniae* and aminopencillin use for uncomoplicated CAP and reported clindamycin resistance and clindamycin selection for PPE. To identify variables for inclusion in the model, we first examined the bivariate associations between independent and dependent variables. The independent variables considered for inclusion in the models were hospital type (freestanding children's, children's hospital within a hospital or pediatric ward); whether or not the hospital has housestaff; geographic region; presence of an ASP; presence of a CAP clinical guideline; and reported resistance levels for penicillin, MRSA and clindamycin. The dependent variables for the two models were aminopenicillins for uncomplicated CAP and clindamycin for PPE. In the final models we included variables with a bivariate association of p <0.2. For certain variables where we hypothesized that a relationship might exist (e.g. ASP and CAP clinical guideline) we tested the model outcome by forcing the variable into the model even if the bivariate association was below 0.2. All analyses were conducted using STATA 11 (STATA CORP, College Station, TX).

## Results

We received responses from 148 out of 259 members for an overall response rate of 57%. We found no differences between respondents and non-respondents in terms of their employment settings, the region of the country where they practiced, or their total years of experience. Seven respondents did not provide inpatient clinical care, leaving 141 respondents for subsequent analysis. Among these, 48% reported having an ASP at their institution and 34% reported having a clinical pathway or guideline for CAP.

### Antibiotic regimens for uncomplicated CAP

There was considerable variation in the categories of recommended regimens for uncomplicated CAP (Table 1). Data were available from all 141 respondents. Most respondents (95%) recommended a beta-lactam, although they were divided among those recommending a beta-lactam alone and those recommending a beta-lactam in combination with another antibiotic category. Azithromycin was recommended only in the context of combination therapy (Table 1). An anti-MRSA regimen was recommended in 20% of cases, typically in combination with beta-lactam therapy.

**Table 1:** Empiric antibiotic regimens recommended for uncomplicated pneumonia and PPE.

	Uncomplicated(%, N = 141)	PPE(%, N = 140)
Beta-lactam Alone	77 (55)	10 (7)
Beta-lactam in combination	57 (40)	127 (90)
*Beta lactam+macrolide*	*36 (26)*	*4 (3)*
*Beta lactam+anti-mrsa*	*20 (14)*	*99 (71)*
*Beta lactam+anti-mrsa+macrolide*	*1 (1)*	*24 (17)*
Anti-mrsa alone	5 (4)	1(1)
Anti-mrsa+macrolide	2 (1)	2 (1)

PPE, parapneumonic empyema.

MRSA, methicillin-resistant *S. aureus*.

### Antibiotic regimens for PPE

For PPE, there was similar variability in terms of the antibiotic categories included in the recommended regimens (Table 1); 1 respondent did not include antibiotic recommendations for PPE, leaving 140 respondents for analysis. Most (90%; 127/140) recommended including an anti-MRSA antibiotic in their empiric regimen, typically in combination with a beta-lactam antibiotic with or without concomitant macrolide therapy. Only 7% (10/140) recommended a beta-lactam alone and 3% (4/140) recommended a beta-lactam plus azithromycin.

### Antibiotic selection within categories

In addition to variation in the antibiotic categories that were recommended, there was variation within categories in terms of the specific agents recommended. For beta-lactams, 74% (105/141) of respondents recommended a cephalosporin for uncomplicated CAP while 95% (133/140) recommended a cephalosporin for PPE. Aminopencillins were recommended for uncomplicated CAP by 25% (35/141) of respondents. For anti-MRSA agents, further variation was noted. For uncomplicated cases, clindamycin was recommended by 86% (24/28) of respondents and vancomycin by 14% (4/28) of those recommending this category. For PPE, clindamycin was included in the recommended anti-MRSA regimen by 44% (55/126) of respondents, vancomycin by 57% (72/126) and linezolid by 4% (5/126). This sums to >100% due to 6 respondents recommending multiple anti-MRSA agents concurrently.

### Duration of therapy

**Figure 1:**
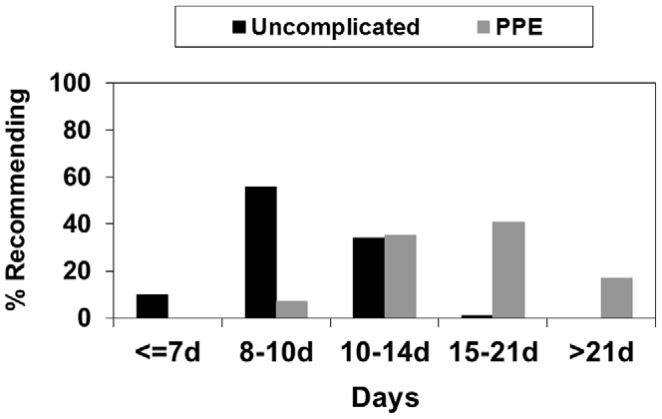
Recommended duration of therapy for uncomplicated CAP (N = 140) and PPE (N = 138). CAP, community-acquired pneumonia; PPE, parapneumonic empyema.

Overall, respondents generally recommended longer duration for PPE than for uncomplicated cases (Figure 1). For uncomplicated cases, 140 respondents provided data for duration and only 10% (14/140) recommended a duration of 7 days or less. The most common duration was 8–10 days, recommended by 56% (78/140). For PPE, 138 respondents provided data and 35% (48/138) recommended 11–14 days, 41% (57/138) recommended 15–21 days and 17% (24/138) recommended >21 days. We found no associations between treatment duration and antibiotic regimens, reported MRSA prevalence, the presence of a clinical guideline or ASP or years of experience for either uncomplicated CAP or PPE.

### Relationship between reported antibiotic resistance and antibiotic recommendations

#### Aminopenicillins for uncomplicated CAP

127 respondents provided data on *S. pneumoniae* resistance. Among these, 27 (21%) recommended either ampicillin or ampicillin/sulbactam as the sole agent for uncomplicated CAP while 100 recommended another regimen. The percentage of respondents recommending aminopenicillins did not increase as the reported prevalence of penicillin non-susceptible pneumococci decreased. Aminopenicillins were recommended in 26% of cases where the reported prevalence of penicillin non-susceptible pneumococci was <10% and 25% of cases where the prevalence of penicillin non-susceptible pneumococci was 26%–50% (p-value for trend = 0.46). Only 4 respondents reported prevalence >50% for *S. pneumoniae* non-susceptibility and none recommended aminopenicillins. In multivariable analysis, based on bivariate associations, geographic region, reported prevalence of penicillin non-susceptible pneumococci, presence of ASP and CAP clinical guideline were included in the model. None of these variables were independently associated with recommending aminopenicillins.

#### Anti-MRSA agents

**Figure 2:**
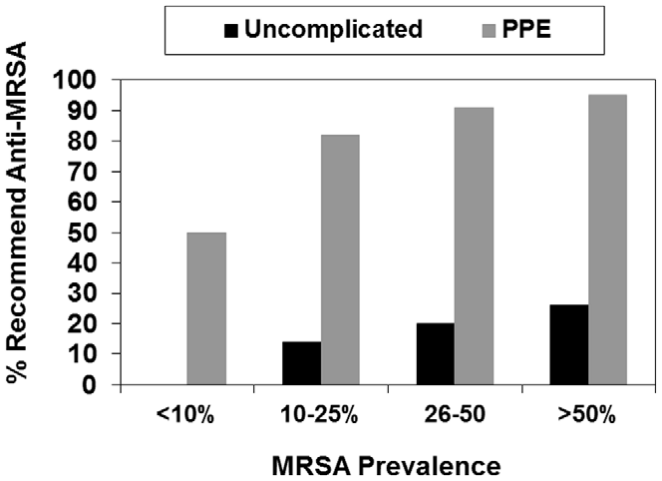
Percentage of respondents who recommended an anti-MRSA agent for uncomplicated CAP (N = 135) and PPE (N = 134) across a range of reported MRSA prevalence levels in their community. MRSA, methicillin-resistant *S. aureus*; CAP, community-acquired pneumonia; PPE, parapneumonic empyema.

There were 135 respondents who provided data on MRSA prevalence and empiric antibiotic recommendations for uncomplicated CAP and 134 for PPE. In contrast to penicillin non-susceptible pneumococci, we observed an increasing percentage of respondents who recommended anti-MRSA agents across the increasing levels of reported MRSA prevalence for uncomplicated CAP and PPE (Figure 2). For uncomplicated CAP, the percentage increased from 0% of respondents recommending anti-MRSA agents who reported MRSA prevalence <10% to 26% of those who reported MRSA prevalence >50% (p trend = 0.14). For PPE, the percentage increased from 50% of respondents who reported MRSA prevalence <10% to 94% of respondents who reported MRSA prevalence >50% (p trend = 0.01).

### Selection of clindamycin for an anti-MRSA agent

**Figure 3:**
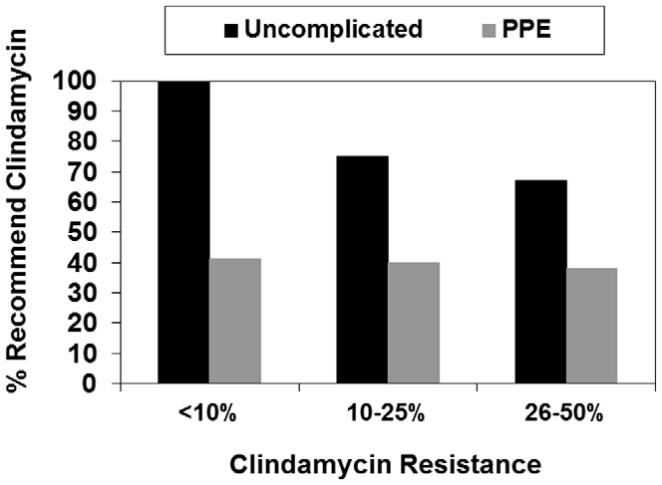
Percentage of respondents who recommended clindamycin as an anti-MRSA agent for uncomplicated CAP (N = 27) and PPE (N = 117) across a range of reported clindamycin resistance levels. MRSA, methicillin-resistant *S. aureus*; CAP, community-acquired pneumonia; PPE, parapneumonic empyema.

There were 27 respondents who selected an anti-MRSA agent for uncomplicated CAP and provided data on clindamycin resistance and 117 for PPE. Overall, we found no direct relationship between the selection of clindamycin as an anti-MRSA agent and the reported level of clindamycin resistance (Figure 3). For uncomplicated CAP, examining this relationship is somewhat limited due to the fact that clindamycin was the anti-MRSA agent recommended by 86% of respondents. There was a trend towards a decline in the percentage of respondents who selected clindamycin as reported clindamycin resistance increased, declining from 100% among those reporting clindamycin resistance <10% to 67% among those reporting clindamycin resistance 25–50% (p trend = 0.06), however, selection of clindamycin remained high overall. For PPE, the percentage recommending clindamycin as the anti-MRSA agent was unchanged as reported clindamycin resistance increased, ranging from 38%–41% across all levels (p trend = 0.86).

Because reported clindamycin resistance did not seem to have a strong influence on selection of clindamycin versus another anti-MRSA agent, especially for PPE, we developed a multivariable logistic model to assess whether any other factors were associated specifically with recommending clindamycin for PPE. Based on bivariate associations, the variables included in the model included reported MRSA prevalence and clindamycin resistance, practicing at a teaching hospital, the presence of a clinical pathway for pneumonia and the number of years of clinical experience in infectious disease. The only factor that was independently associated with recommending clindamycin was years of experience. Compared with those with >15 years of experience, the odds of recommending clindamcyin as an anti-MRSA agent for PPE (as opposed to vancomycin or linezolid) was substantially greater for those with <5 years (OR 7.8; 95% CI 1.9–31.3) and 5–15 years (OR 6.4; 95% CI 1.6–25.3). These results were unchanged when presence of an ASP was forced into the model.

## Discussion

Our study has two important findings. First, we found evidence that substantial variation exists in the recommendations for CAP management from pediatric infectious diseases consultants, including the empiric antibiotic regimens and duration of therapy. Second, we found that differences in reported local antibiotic resistance patterns do not fully account for variations in antibiotic prescribing recommendations.

There are currently no national clinical guidelines for pediatric CAP, although 33% of our respondents indicated that they had a guideline established at their hospital. While the existence of a local guideline could reduce variation within an individual institution, the lack of association between the presence of a guideline and any of the practice patterns we examined (e.g. aminopenicillins for uncomplicated CAP) suggests that existing CAP guidelines are not uniform. Some of the variation we observed may reflect local factors that differ between hospitals including resistance patterns or formulary differences. It is likely, however, that much of the variation in terms of recommended antibiotics and treatment duration simply reflects a lack of consensus, due in part to limited evidence. A recently developed CAP clinical guideline from the Infectious Diseases Society of America (IDSA) has the potential to improve clinical practice and reduce variation.

CAP is a model condition for the development of clinical guidelines and ASP interventions considering that it is a common condition, has geographic variation in etiology and resistance and the potential for significant practice pattern variation. Empiric prescribing practices need to be individualized to account for each institution's local antibiotic resistance patterns. Additionally, treatment of CAP, especially PPE, is made more challenging by the fact that cultures are frequently negative, although newer molecular methods are promising [16]. The development and dissemination of clinical guidelines as well as antibiograms to assist with clinical decision making are important ASP functions [14]. Although antibiograms that are tailored to specific patient populations with specific conditions (e.g. pneumonia) have the potential to improve empiric antibiotic prescribing, they may not be easily accessible or widely used [17]. Furthermore, ASPs and guidelines can emphasize the importance of viral testing for patients with CAP, as certain viral infections such as influenza are associated with higher rates of CAP complications and differences in the etiology of bacterial co-infection [18]. Previous studies indicate that many pediatric institutions do not have ASPs and that existing ASPs have substantial deficiencies [15]. Improving the extent to which unit and condition specific antibiograms are made accessible to providers is a key area for ASP to support front-line prescribing physicians [11], [12]. Our findings that ASPs and CAP guidelines were not associated with differences in treatment recommendations suggest that opportunities exist for these interventions to focus on enhancing the treatment of CAP with narrower spectrum antibiotic alternatives (e.g. ampicillin) and the extent to which treatment recommendations match local resistance patterns.

We were surprised to find no relationship between recommending clindamycin for PPE and reported clindamycin resistance. In particular, many respondents noted that they would recommend clindamycin despite noting relatively high levels of resistance in their community. There are several potential explanations for this result. A lack of reliable susceptibility data from antibiograms is one possibility. Another is that other factors favoring clindamycin relative to vancomycin, such as the availability of an oral formulation and that therapeutic drug monitoring is not necessary, are more important drivers for ID consultants' recommendations. A previous study suggested that ID consultants do not rank antibiotic resistance among the most important factors influencing their prescribing decisions [19]. It may also be that some respondents who prescribe clindamycin in settings where resistance was reportedly high feel that *S. aureus* is an uncommon cause of complicated CAP and thus the risk of a susceptibility mismatch is low. We did, however, observe a relationship between higher reported MRSA prevalence and more recommendations for anti-MRSA agents, indicating that respondents accounted for it as an important pathogen.

Unlike the association between antibiotic selection and MRSA prevalence, we did not observe a relationship between antibiotic selection and resistance in the case of aminopenicillins and the reported prevalence of penicillin non-susceptible *S. pneumonia*e. As with selecting clindamycin for MRSA, other factors besides resistance may contribute to physician preferences. In this instance, some physicians may find aminopenicillins less attractive than cephalosporins because of greater dosing frequency [20], even with low prevalence of penicillin non-susceptible *S. pneumoniae*. At the same time, evidence supports the use of aminopenicillins for treatment of CAP, even in the setting of relative resistance [21], [22], [23], which may explain why some respondents were comfortable prescribing this class with in this context.

We found that respondents with fewer years of clinical experience were far more likely to prescribe clindamycin for PPE than those with more clinical experience. A potential explanation for this finding is that respondents who completed training more recently may have more readily adopted practice patterns reflecting the emergence of the USA300 strain of community-associated MRSA, which is frequently susceptible to clindamycin [24].

We are not aware of any other studies that have explicitly examined the relationship between reported antibiotic resistance and antibiotic selection. Our findings indicate that many factors contribute to antibiotic selection, and perceived levels of antibiotic resistance is not necessarily the primary one. Nonetheless, our findings with respect to recommendations for clindamycin do raise some concerns. Use of clindamycin for treatment of pediatric MRSA infections has increased dramatically nationwide [25]. Because there may be seasonal, institutional and geographic variation in the organisms that cause uncomplicated and complicated CAP, combined with variation in resistance patterns, it is imperative that physicians have access to updated epidemiologic data for their community. Indeed, over 80% of our respondents indicated that they desired more information about local antibiotic resistance rates to assist in empiric prescribing decisions.

We observed variation in recommendations for macrolides. Monotherapy with a macrolide was not recommended by any respondents, even for uncomplicated CAP, presumably as a reflection of *S. pneumonia*e resistance. Although IDSA guidelines for the treatment of adult CAP recommend empiric therapy targeting atypical pathogens [26], evidence favoring use of macrolides to target these organisms (especially *Mycoplasma pneumoniae*) for treatment of pediatric CAP is not convincing [27].

There are several limitations to our study. Because the data are derived from a survey and not from an administrative database or chart review, the respondents' reported practices might not match their actual practices. Infectious diseases physicians are not usually the primary prescribers for children with CAP, however, they do frequently contribute to guidelines and local practice consensus. Physicians may have different antibiotic prescribing practices for pediatric CAP for patients in different age groups (e.g. <1–5 years and >5 years) which was not captured by this survey. Our analysis of resistance was based on respondents' report rather than antibiogram data and thus may not reflect actual epidemiology. However, the relationship between what respondents believe the resistance patterns to be to their and their practice patterns is likely a more important driver of practice.

We found that substantial variation exists among pediatric infectious disease consultants in their recommendations for empiric antibiotic regimens for pneumonia and that this variation is only partly related to reported local antibiotic resistance patterns. One area highlighted by our findings is that greater dissemination improvements to resources about the epidemiology of local resistance patterns may assist physicians in their selection of empiric antibiotic regimens for CAP or other conditions where antibiotic resistance is common.
